# The American Dental Association

**Published:** 1883-09

**Authors:** 


### THE AMERICAN DENTAL ASSOCIATION.

The twenty-third annual meeting of this Society was chiefly remarkable for that in which it should take least pride. It would be much more agreeable, from many considerations, to give nothing but commendation, or in silence to pass over the faults exhibited; but a journal that professes to speak for the whole profession owes its first duty to its readers, and so plain words are best.

There was no lack of numbers present, nor was there anything of moment to mar the harmony of the proceedings. But the general tone of the meeting was not what the representative association of so large a profession, one which embodies so much of professional learning and personal intelligence, should exhibit. Some of the papers read showed thoughtful consideration and careful research, but the most of the few presented were far below the standard of a national congress of dentists. Nor were the discussions as a whole what they might have been. Too many members talk, not because they have something to say, but to exhibit themselves; to hear their own voice, and to be put upon the record as having spoken, even if it were nothing but platitudes. It was really painful at times to see men buffet and belabor their brains, when their pump perceptibly "sucked air." Many of those who occupied valuable time showed that they had very little appreciation of the subject under consideration. But that is a fault that is not confined to the A. D. A., as all attendants upon medical and dental meetings will testify. When really scientific matters were discussed there seemed to be an impatience manifested, and members plainly indicated their lack of interest, and their desire to return to general subjects, or to fly the track entirely and commence the everlasting topics of ethics, by-laws, etc. When the representative dentists of America get together they should busy themselves with something more weighty than matters of detail.

The subject of education always occupies much time, and it is occasionally amusing to see the class of dentists who are the most clamorous for an advanced standard, and who most severely take our colleges to task for their shortcomings. Some of these purist critics are men without any degree at all, and some are quite incapable of constructing a grammatical English sentence. Yet they are among the most vociferous in their outcry for a "higher standard." Our colleges doubtless might be made much better, but they are fully up to the present status of the profession as a whole, and deserve commendation for what they have accomplished, rather than indiscriminate vituperation and harsh criticism, especially if it comes from those least competent to sit as judges.

The Association is afflicted with a class of politicians, who apparently attend its meetings for the mere pleasure of pulling the wires. Most of the older members go there to exchange views upon matters of professional interest, and are quite willing to leave to partisans the self-elected task of running the "machine." These men are useful in their way, and certainly some one must look after routine matters; but when they interfere with scientific affairs it is time that a halt was called.

One thing transpired at Niagara that has grieved many, and we cannot but think has tarnished the good name of the Association. We do not propose to set up as a censor of professional morals, nor do we make any personal charges whatever, for we were not present when the action was taken, and have no knowledge therefore concerning who was responsible for what, as we look at the matter, was an outrage upon the rights of the majority, and a blot upon the fair fame of the Society.

Last year the Association announced that it would award a prize to the author of the best essay upon a certain subject, provided it come up to a certain standard. It appointed a prize committee to whom it delegated its authority, and it empowered them to act as judges in awarding the prize. At the Niagara meeting this committee reported that a paper had been presented which conformed to the conditions, came up to the designated standard, and they therefore awarded the prize to its author. The sole question before the Society then was: shall the report be accepted? If it were, according to all precedent and good usage, the matter was ended. If the report were rejected, the Association had done something which it was quite competent to do, but which could not be done without marked discourtesy to its committee, and great injustice to those most nearly concerned. In a very full session, with nearly all the members present, the report after due consideration was adopted with but one dissentient voice. This was well. But just at the close of the last session, held after most of the trains which were to carry members to their homes had gone, and when but a very few, scarce a tenth of the members were present, this action was reversed, and the paper referred back to the committee for another year. Of course every one knows that no progressive man, in the present wonderful growth of professional knowledge, would be willing to have presented as his views something that he had written more than a year before, and therefore this was an act of great injustice to the author. Besides, the Society had pledged itself that under certain conditions it would award a prize. Those conditions were fulfilled, according to the report of its own accredited judges, when a mere minority assumed to override the expressed opinion of the members, repudiate a just obligation, violate the pledge made the previous year, and thus tarnish the Honor of an association of honorable men. When reasoned with concerning this breach of good faith, the answer was returned, that the majority should then have staid until the close of the proceedings. And is the A. D. A. then, but a caucus of demagogues? Is it but a smart trick to overrule by sharp practice the will of the majority? Are not the members men whom it is safe to trust in the dark, or are they people who cannot be depended upon lest at an unguarded moment they spring a trap upon the unwary?

We do not think such an answer will be accepted on the part of those then present. It would rather appear that, with most of them, it was an act thoughtlessly done, without due consideration of the consequences, but unless their action be, at the next meeting, repudiated, the A. D. A. will scarce be worthy the consideration which ought to be accorded to it.

If this action be due to some of those exceedingly sharp men who forget that the American Dental Association is composed of gentlemen, and is not a political caucus which it is a smart thing to manipulate; if they imagine that they have any prescriptive right to dictate a policy for their compeers to follow, they would do well to consider the story of the historical fly. We will tell the tale for the benefit of whom it may concern.

A fly upon the rim of a carriage wheel was complacently contemplating its own importance and the surrounding scenery. "See" it said "how everything in nature revolves about *me*." Just then the wheel struck a stone, and the concussion seriously disturbed it. equanimity. "What a convulsion of nature," was its comment. "If this occurs again I will get off, and then what will become of a Universe deprived of its centre."

What we have said we hope will not be misunderstood. It is spoken in no cynical, carping mood, but because we love the American Dental Association, and are loyal to it, and have at heart its best good. We desire it to be worthy the name of the representative association of America. We thought that this journal ought to give voice to the expression of many dentists, and therefore we have written this article.

We had it in mind to speak of the *good* things of the meeting, of the rare good-fellowship exhibited, and the kindly personal feeling manifested by members toward each other, but we have taken it all out in growling, and there is no space for felicitation.

